# Genus *Klebsiella*: Infections Encountered in a General Surgery Department and Antimicrobial Drugs Susceptibility Patterns

**DOI:** 10.3390/microorganisms14040773

**Published:** 2026-03-28

**Authors:** Sergiu-Ciprian Matei, Justin Horia Lăpușan, Ana-Maria Ungureanu, Edida Maghet, Vlad Meche, Marcel Berceanu Vaduva, Claudia Livia Stanga, Mihaela Valcovici, Abhinav Sharma, Nilima Rajpal Kundnani

**Affiliations:** 1 Abdominal Surgery and Phlebology Research Center, “Victor Babes” University of Medicine and Pharmacy, 300041 Timisoara, Romania; matei.sergiu@umft.ro; 2 1st Surgical Department, “Pius Brinzeu” Emergency County Hospital, 300723 Timisoara, Romania; 3 Faculty of Medicine, “Victor Babes” University of Medicine and Pharmacy, 300041 Timisoara, Romania; 4 Department XV, Clinic of Radiology and Medical Imaging, “Victor Babes” University of Medicine and Pharmacy, Eftimie Murgu Square, No. 2, 300041 Timisoara, Romania; 5 Department of Radiology and Medical Imaging, “Pius Brinzeu” Emergency County Hospital, 300723 Timisoara, Romania; 6 Faculty of Dental Medicine, “Victor Babes” University of Medicine and Pharmacy, 300041 Timisoara, Romania; 7 Doctoral School, “Victor Babes” University of Medicine and Pharmacy, 300041 Timisoara, Romania; 8 Department XVI—Orthopedics-Traumatology—I, Urology and Medical Imaging, “Victor Babes” University of Medicine and Pharmacy, 300041 Timisoara, Romania; 9 Department XIV—Microbiology, “Victor Babes” University of Medicine and Pharmacy, 300041 Timisoara, Romania; 10 Department VI—Cardiology, “Victor Babes” University of Medicine and Pharmacy, 300041 Timisoara, Romania; 11 Research Centre of Timisoara, Institute of Cardiovascular Diseases, “Victor Babes” University of Medicine and Pharmacy, 300041 Timisoara, Romania

**Keywords:** *Klebsiella pneumoniae*, infections, urinary catheters nosocomial, intra-abdominal abscess

## Abstract

*Klebsiella* species, particularly *Klebsiella pneumoniae*, are among the most frequently isolated Gram-negative pathogens in surgical departments, associated with a growing trend in multidrug resistance. To identify the types of infections caused by *Klebsiella* spp. in a general surgery department and to analyze their antimicrobial susceptibility patterns. This retrospective observational study includes bacteriological cultures collected from surgical inpatients between October 2016 and December 2024. Only cases with confirmed *Klebsiella* spp. isolation were included. Specimen types, infection categories, and antibiotic susceptibility profiles were extracted and analyzed. A total of 138 *Klebsiella*-positive cultures were identified. Clinical characteristics were analyzed in 38 patients with complete records. The most common infection types included surgical site infections (SSIs), intra-abdominal infections, and biliary tract infections. Sensitivity was highest to carbapenems, while marked resistance was observed to ampicillin-sulbactam and third-generation cephalosporins. Some isolates exhibited ESBL or carbapenemase-producing phenotypes. Reported colistin non-susceptibility was elevated in our cohort; however, these results should be interpreted cautiously because the reference broth microdilution method was not systematically documented. The findings underscore the importance of local surveillance of *Klebsiella* spp. in surgical settings to info rm empirical treatment and control the spread of multidrug-resistant organisms.

## 1. Introduction

*Klebsiella pneumoniae* is a prominent member of the Enterobacteriaceae family and a leading cause of nosocomial infections, particularly in high-risk hospital units such as general surgery and intensive care. It is frequently isolated from surgical wounds, intra-abdominal abscesses, biliary secretions, and urinary catheters. Its capacity to cause both community-acquired and hospital-acquired infections, including pneumonia, bloodstream infections, and urinary tract infections, has made it a significant public health threat.

Over recent decades, *Klebsiella* spp. have demonstrated a concerning ability to acquire and disseminate antimicrobial resistance determinants. The emergence of extended-spectrum beta-lactamase (ESBL) producing strains has led to reduced efficacy of third-generation cephalosporins, while the rise in carbapenem-resistant *Klebsiella pneumoniae* (CRKP) has further narrowed therapeutic options. Infections caused by these resistant strains are associated with increased morbidity, mortality, length of hospital stay, and healthcare costs.

In surgical patients, *Klebsiella* spp. infections present unique challenges due to frequent exposure to invasive procedures, foreign materials, and repeated antimicrobial courses. These factors contribute to both increased infection risk and selective pressure favoring multidrug-resistant strains. General surgery wards, therefore, represent a critical interface between community-acquired pathogens and highly resistant hospital-adapted clones, proving the importance of unit-specific epidemiological surveillance.

According to a multicenter surveillance report, *Klebsiella pneumoniae* is among the top three Gram-negative pathogens in surgical site infections, with increasing resistance to third-generation cephalosporins and fluoroquinolones [1]. This finding is echoed across many healthcare systems globally, where *Klebsiella* is a major contributor to hospital-acquired infections, particularly in resource-limited environments where infection control and antibiotic stewardship may be suboptimal.

Geographic variability further complicates the issue, with resistance profiles differing widely between regions. A retrospective analysis from southern Italy found that over 40% of *Klebsiella pneumoniae* isolates were resistant to at least one carbapenem, and the proportion of ESBL-producing isolates increased annually [2]. Similarly, a study from a Saudi tertiary hospital revealed that surgical patients exhibited higher rates of multidrug-resistant (MDR) *Klebsiella* compared to medical patients, with resistance to piperacillin-tazobactam and cefepime exceeding 50% [3].

Multiple mechanisms underpin *Klebsiella*’s resistance, including plasmid-mediated β-lactamase production, efflux pump overexpression, and porin mutations. These capabilities enable rapid adaptation and transmission within hospital ecosystems, especially when compounded by frequent antibiotic exposure and invasive procedures in surgical units.

Given these challenges, ongoing local surveillance is essential to guide empirical therapy and infection control strategies. Our study seeks to contribute to this growing body of knowledge by retrospectively analyzing *Klebsiella*-associated infections in a general surgery department. Specifically, we aim to characterize infection types, specimen sources, and antimicrobial susceptibility profiles, with an emphasis on identifying resistance trends relevant to clinical practice.

## 2. Materials and Methods

### 2.1. Study Design

This retrospective observational study was conducted in the General Surgery Department of the ‘Pius Brînzeu’ Emergency County Hospital, Timișoara, Romania. The study covered a 8-years and 2 months period, from 1 October 2016, to 31 December 2024. Patients included in the analysis were those admitted to the department for surgical treatment or care during this period. Specimens were collected continuously from October 2016 to December 2024, with 138 *Klebsiella*-positive cultures identified during this period.

### 2.2. Inclusion Criteria

All inpatients with laboratory-confirmed cultures positive for *Klebsiella* species were included. Only the first isolate per patient per infection site was considered to avoid duplication. Patients with incomplete microbiological records or whose cultures grew *Klebsiella* in the absence of clinical signs of infection were excluded.

While all eligible *Klebsiella*-positive cultures were included in microbiological analyses (*n* = 138), demographic characteristics and clinical outcomes were analyzed only in patients with complete clinical documentation available in the electronic medical records (*n* = 38).

The statistical unit for antimicrobial susceptibility analyses was the isolate (*n* = 138). The statistical unit for demographic and outcome analyses was the patient with complete clinical documentation (*n* = 38).

The observed number of cases reflects strict inclusion criteria, as only clinically significant, culture-confirmed *Klebsiella* infections were included, while screening cultures, colonization, and duplicate isolates were excluded.

An overview of the *Klebsiella* isolates included in the study, stratified by specimen type, species, and infection category, is provided in Supplementary Table S1.

### 2.3. Data Collection

Data were extracted retrospectively from hospital electronic medical records and microbiology lab information systems. The following variables were collected:

- Patient identifier (anonymized);
- Admission and detection dates;
- Method of pathogen isolation;
- Sample type and infection site;
- Identified bacterial species;
- Antibiotic resistance profile (qualitative and narrative format).

The data were exported from Microsoft Excel 365 and converted into standardized tables for analysis.

### 2.4. Microbiological Procedures

Clinical specimens were processed using standard microbiological protocols, including inoculation on selective and non-selective media followed by incubation and biochemical identification of pathogens. Antibiotic susceptibility testing (AST) was performed using automated methods according to Clinical and Laboratory Standards Institute (CLSI) guidelines. Interpretations were categorized as susceptible (S), intermediate (I), or resistant (R) based on MIC breakpoints. Extended-spectrum beta-lactamase (ESBL) production was confirmed using combination disk tests. Carbapenemase-producing isolates were flagged based on phenotypic confirmatory tests, such as modified Hodge test or carbapenem inactivation method (CIM), if performed. Colistin susceptibility testing was performed as part of routine laboratory workflow; however, broth microdilution, the reference method recommended by EUCAST, was not systematically documented for all isolates.

Identification of *Klebsiella* species was performed using routine biochemical and automated identification systems available in the hospital laboratory at the time of isolation. Antimicrobial susceptibility testing results were interpreted according to the CLSI M100 guidelines in use in the hospital laboratory at the time of testing. Because CLSI breakpoints may vary across guideline editions, this variability is acknowledged as a limitation of the retrospective design. Phenotypic confirmatory tests for ESBL or carbapenemase production were performed when clinically indicated, but not systematically for all isolates.

Colistin susceptibility results were extracted from routine laboratory AST reports. However, the specific colistin testing methodology (e.g., broth microdilution versus automated systems) was not systematically recorded in the available retrospective documentation for all isolates.

### 2.5. Specimen Types and Infection Classification

The majority of specimens were obtained from:

- Wound secretions and swabs;
- Peritoneal fluid (drainage or aspirate);
- Biliary secretions or gallbladder fluid;
- Abscess material (e.g., hepatic, parietal).

Infections were categorized based on clinical and microbiological criteria into the following types:

- Surgical site infections (SSIs);
- Intra-abdominal infections;
- Biliary tract infections;
- Liver abscesses;
- Polymicrobial or undetermined infections.

Infection categories were assigned based on clinical presentation, imaging findings, and microbiological results, in line with commonly accepted CDC and ECDC definitions for healthcare-associated infections, adapted to local clinical practice.

### 2.6. Data Analysis

Data processing and analysis were conducted using Microsoft Excel 365 for initial tabulation and formatting, and SPSS-31 for data cleaning, filtering, statistical summaries, and visualization.

Descriptive statistics were used to summarize categorical variables (infection types, antibiotic susceptibility patterns). Frequencies and proportions were calculated to determine the distribution of *Klebsiella* isolates across infection types and resistance profiles.

No formal hypothesis testing was pre-specified, given the descriptive and exploratory nature of the study. Statistical analyses were intended to characterize local epidemiology rather than infer causality or risk factors. Continuous variables were summarized using medians and ranges where applicable. No inferential statistical testing was planned, as the study aimed to provide a descriptive overview of local epidemiology rather than to test predefined hypotheses.

Resistance scores were calculated as the proportion of isolates classified as resistant among those tested for each antibiotic.

Antimicrobial susceptibility testing results were analyzed at the isolate level. For each antimicrobial agent, resistance proportions were calculated using the number of isolates tested as the denominator. Aggregate resistance phenotype data are presented in Supplementary Table S2.

A complete isolate-level dataset including specimen type, infection category, species identification, and antimicrobial susceptibility profiles for each isolate is provided in Supplementary Table S3.

### 2.7. Ethical Considerations

Before the commencement of the study, Ethical approval for this study was granted by the Review Board of the University of Medicine and Pharmacy of Timisoara affiliated County Emergency Hospital “Pius Branzeu” (Approval number: 528/04.02.2025) based on the Helsinki Declaration. Patient identities were anonymized at the point of data extraction to ensure confidentiality.

### 2.8. Study Limitations

This investigation has several limitations:

- A single-center design limits the generalizability of findings to other surgical units or geographic regions.
- Retrospective data may include missing or incomplete records, especially in susceptibility reporting.
- Free-text susceptibility fields required manual parsing, which could introduce classification bias or errors.
- Phenotypic-only resistance screening: Molecular confirmation of ESBL or carbapenemase genes was not performed.

## 3. Results

**Figure 1:**
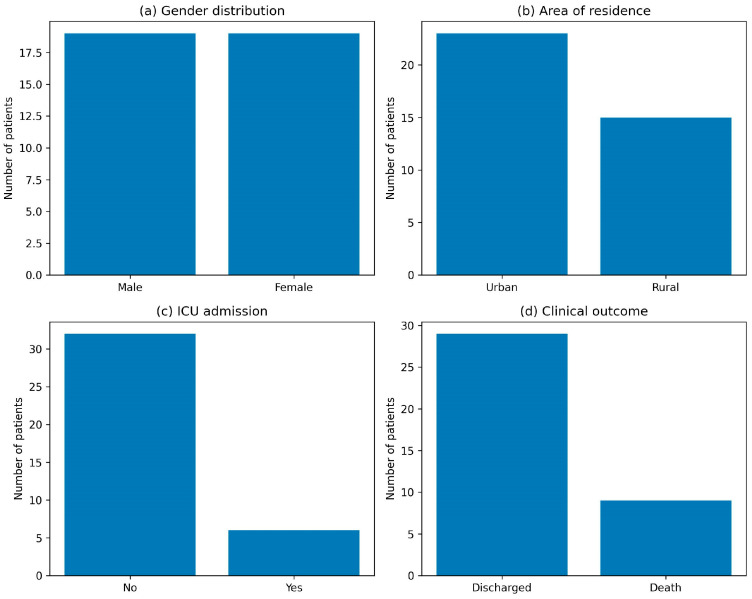
Distribuition chart based on different parameters. Gender Distribution: An Even Distribution Was Recorded, with 50% Male (*n* = 19) and 50% Female (*n* = 19) (**a**). Distribuition based on Native Environment: Patients Primarily Originated from Urban Environments: Urban (U): 60.5% (*n* = 23); Rural (R): 39.5% (*n* = 15) (**b**). Distribuition based on ICU Hospitalization: Only a Minority Required Intensive Care, Suggesting That Not All Klebsiella Infections Progressed to Critical Illness: No ICU admission: 84.2% (*n* = 32), ICU admission: 15.8% (*n* = 6) (**c**). And based on clinical outcome: Outcomes Were Favorable in the Majority of Cases, discharged (E): 76.3% (*n* = 29), death (D): 23.7% (*n* = 9) (**d**).

Out of 1341 bacteriological exams performed during the time-set period, 138 *Klebsiella* species infections were noted (10.29%). The statistical unit for antimicrobial susceptibility analyses was the isolate, while the statistical unit for demographic and clinical variables was the patient. All analysis was performed on the subgroup of patients with complete clinical data (*n* = 38). Among patients with complete clinical data (*n* = 38), the following distributions were observed (Figure 1):

### 3.1. Antibiotic Resistance Patterns

**Figure 2:**
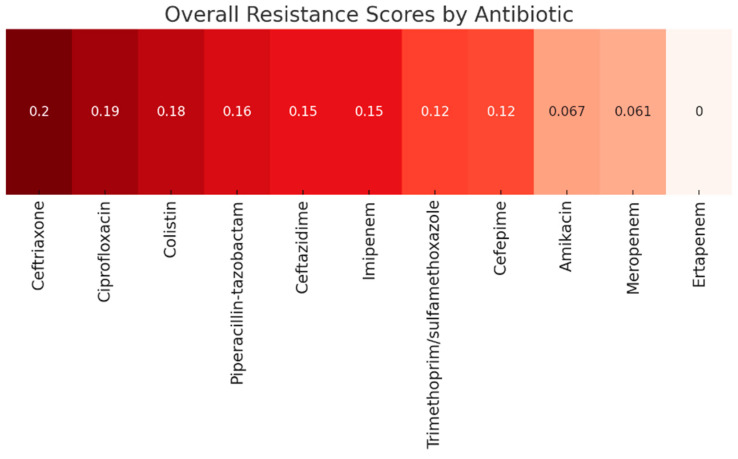
Overall Resistance Scores by Antibiotic. Resistance scores represent the proportion of resistant isolates for each antibiotic (e.g., 0.20 corresponds to 20% resistance).

Antimicrobial resistance analyses were performed on all *Klebsiella*-positive cultures identified during the study period (*n* = 138). Resistance profiles varied, but several key patterns emerged. High resistance was observed to ampicillin, piperacillin, and certain third-generation cephalosporins. Some isolates retained susceptibility to amikacin, aztreonam, and carbapenems. Multiple phenotypes indicated extended-spectrum beta-lactamase (ESBL) activity. Below is a heatmap illustrating the pooled resistance profile across all *Klebsiella* isolates, providing an overall overview of susceptibility patterns (Figure 2).

The heatmap reveals a clear gradient in resistance among the tested antibiotics for *Klebsiella* isolates from surgical patients. Ceftriaxone, Ciprofloxacin, and Colistin showed a comparatively high non-susceptibility signal in the pooled data, highlighting a worrying trend toward reduced effectiveness of third-generation cephalosporins and certain last-resort agents like colistin. This finding is clinically significant because colistin is often reserved for MDR Gram-negative infections; therefore, elevated non-susceptibility may further restrict salvage options in severe surgical infections. Resistance to Piperacillin–tazobactam and Ceftazidime also shows the impact of extended-spectrum beta-lactamase (ESBL) activity, as noted in the study.

On the other hand, carbapenems (Imipenem (0.15), Meropenem (0.061), and Ertapenem (0.00)) maintain relatively low resistance scores, confirming the fact that they remain among the most effective options against *Klebsiella* spp. in this surgical cohort. Amikacin (0.067) also retains strong activity, making it a valuable choice in severe infections. Trimethoprim-sulfamethoxazole (0.12) and Cefepime (0.12) fall in the mid-range, suggesting partial preservation of activity. However, this shows that caution is needed when relying on them as first-line agents without susceptibility confirmation.

Detailed antimicrobial susceptibility results at the isolate level are presented in Supplementary Table S3, allowing direct linkage between individual isolates, specimen type, and resistance profiles.

### 3.2. Type of Biological Sample

*Klebsiella* isolates were most frequently recovered from:

Surgical wound secretions: 26.3%

Abscess fluid: 23.7%

Peritoneal fluid: 15.8%

Biliary fluid: 15.8%

Resistance rates varied according to specimen type (Table 1). Wound-derived isolates showed higher resistance rates across several antibiotic classes compared with abscess, biliary, and peritoneal fluid isolates, supporting the value of specimen-stratified local antibiograms when selecting empirical therapy.

The distribution of *Klebsiella* isolates by species and specimen type is summarized in Supplementary Table S1.

Aggregate antimicrobial resistance phenotypes among *Klebsiella* isolates are summarized in Supplementary Table S2. Aggregate resistance summaries in Supplementary Table S2 are complemented by isolate-level susceptibility data in Supplementary Table S3.

Detailed isolate-level data are provided in Supplementary Table S3.

### 3.3. Infection Types

The infections were diverse, including surgical site infections, hepatic and perianal abscesses, biliary tract infections and generalized peritonitis.

### 3.4. Overall Antibiotic Resistance

Resistance was highest to colistin, piperacillin-tazobactam, ciprofloxacin, and trimethoprim/sulfamethoxazole. Sensitivity remained better for carbapenems (meropenem, ertapenem, imipenem), amikacin and ceftazidime.

Percentages are calculated using only isolates with available AST results for that antibiotic (tested isolates as denominator). ‘–’ indicates no AST data available for that antibiotic in that specimen category. Antimicrobial resistance data presented in Table 1 are reported at the isolate level, whereas demographic and clinical characteristics refer to the corresponding patient cohort.

### 3.5. Primary Diagnoses

The major surgical diagnoses among these patients included: hepatic abscesses, perforated gastric ulcers, Fournier’s gangrene, complicated colorectal cancers and severe cholecystitis and pancreatitis.

### 3.6. Resistance by Diagnosis

Diagnosis-stratified resistance patterns revealed:

- a higher resistance score in cholecystitis and perianal abscesses;
- more favorable profiles in hepatic abscesses and some retroperitoneal infections.

### 3.7. Labwork

**Figure 3:**
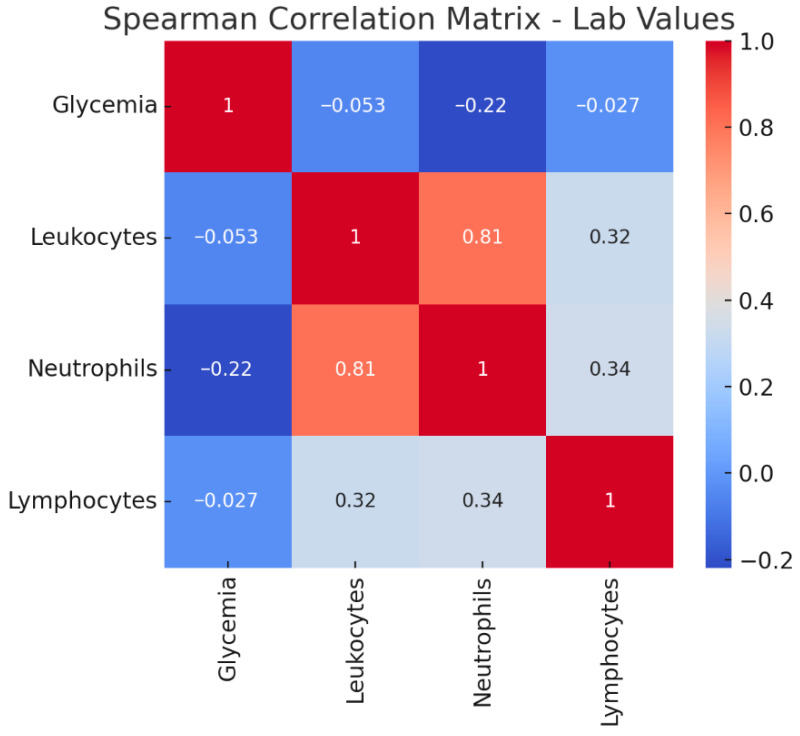
Spearman correlation matrix between lab values.

A Spearman correlation matrix was developed to show the relationships between key laboratory parameters in patients with *Klebsiella* infections (Figure 3).

As expected in bacterial infections, there is a strong positive correlation between leukocyte and neutrophil counts (ρ = 0.81), consistent with a neutrophil-predominant leukocytosis typical of acute bacterial responses. Leukocytes also show a moderate positive correlation with lymphocytes (ρ = 0.32), possibly reflecting cases with mixed inflammatory responses or recovery phases.

Neutrophils demonstrate a similar moderate correlation with lymphocytes (ρ = 0.34), which could indicate overlapping immune activation patterns rather than a purely inverse relationship between the two cell types. Glycemia, however, shows weak and slightly negative correlations with leukocytes (ρ = −0.053), neutrophils (ρ = −0.22), and lymphocytes (ρ = −0.027), suggesting that hyperglycemia in this cohort may be more related to comorbidities or stress response rather than directly tracking with white cell counts.

## 4. Discussion

The present study provides an updated overview of the epidemiology, clinical manifestations, and antimicrobial resistance profiles of *Klebsiella* spp. isolated from patients in a general surgery department. Our findings are consistent with the increasing global burden of multidrug-resistant (MDR) *Klebsiella pneumoniae*, particularly in surgical settings, as documented by several multicentre and regional studies [4,5,6,7]. Surgical site infections (SSIs) represented the most frequent presentation in our cohort, followed by abscesses, intra-abdominal infections, and biliary tract infections, reflecting patterns observed in large SSI surveillance reports [8,9].

The predominance of *K. pneumoniae* in SSIs aligns with the five-year analysis by Lim et al., which identified the organism as one of the leading Gram-negative SSI pathogens, with marked resistance to cephalosporins and fluoroquinolones [10]. Our data similarly demonstrated high non-susceptibility rates to ceftriaxone, ciprofloxacin, and colistin, underscoring the diminishing efficacy of these agents in the surgical setting. The detection of extended-spectrum β-lactamase (ESBL) phenotypes in multiple isolates supports previous observations that ESBL-producing *K. pneumoniae* are increasingly common in surgical populations [11]. ESBL carriage has been associated with worse clinical outcomes and prolonged hospital stays, particularly in ICU patients [12].

Reported colistin non-susceptibility was elevated in our cohort; however, because broth microdilution was not systematically documented, these findings should be interpreted cautiously and confirmed in future prospective studies using reference methods. Because colistin is frequently considered a last-line agent for MDR Gram-negative infections, any rise in non-susceptibility has direct implications for salvage therapy in critically ill surgical patients. This finding warrants local verification, intensified stewardship to reduce selective pressure, and strengthened infection-control measures to prevent dissemination of strains with limited therapeutic options.

Interestingly, only 15.8% of our patients required ICU admission, which may reflect early identification and source control in most cases. However, this contrasts with reports from ICU-based studies where *K. pneumoniae* infections often present with severe sepsis or septic shock [13]. The lower ICU admission rate in our cohort may also be influenced by case selection and the predominance of localized infections rather than bloodstream infections, which carry higher mortality risk [14,15]. Surgical wards may act as reservoirs for resistant *Klebsiella* spp. due to repeated antimicrobial exposure, prolonged hospitalization, and the presence of drains or open wounds. Unlike ICU-focused studies, our cohort reflects a broader surgical population, emphasizing that antimicrobial resistance is not confined to critical care units and requires vigilance across all hospital sectors.

The observed resistance profile, with preserved susceptibility to carbapenems and amikacin, is encouraging in the short term but must be interpreted with caution. Global epidemiological data indicate a steady rise in carbapenem-resistant *K. pneumoniae* (CRKP), with prevalence exceeding 40% in some regions [16,17]. Molecular studies have shown that carbapenem resistance is frequently mediated by the acquisition of *bla*KPC, *bla*NDM, or *bla*OXA-48-like genes, often in combination with porin mutations and efflux pump overexpression [18,19,20]. Once established, CRKP outbreaks can be difficult to control and are associated with mortality rates exceeding 50% in some series [21,22]. The preserved carbapenem susceptibility in our cohort may therefore represent a window of opportunity for intensified infection prevention and antimicrobial stewardship.

Although antimicrobial resistance patterns can change over time, the relatively small number of isolates per year limited the feasibility of meaningful time-stratified analyses. Pooling data across the study period was therefore chosen to provide a robust overview of local resistance patterns. Future studies with larger annual case numbers should evaluate temporal trends, particularly for carbapenem and colistin resistance.

Compared with reports from Western and Southern Europe, the resistance patterns observed in our cohort fall within the intermediate-to-high range for third-generation cephalosporins and fluoroquinolones, while carbapenem resistance remains relatively limited. This contrasts with settings where carbapenem-resistant *Klebsiella* pneumoniae exceeds 30–40%, highlighting important regional variability and the need for localized empirical treatment algorithms.

Biliary tract infections comprised 15.8% of cases, with resistance patterns similar to those described in recent bile culture studies, which reported high ESBL prevalence and reduced susceptibility to piperacillin–tazobactam [23,24]. This is of particular concern in hepatobiliary surgery, where postoperative cholangitis and bile leaks can facilitate rapid dissemination of MDR organisms [25]. Similarly, our finding of higher resistance rates in cholecystitis and perianal abscesses suggests that certain infection sites may act as reservoirs for more resistant *K. pneumoniae* strains, a phenomenon also noted in abdominal SSI reports [26].

Importantly, resistance patterns differed by specimen type in our cohort. This finding highlights a key limitation of pooled resistance summaries and supports the clinical utility of specimen-stratified antibiograms in surgical wards. In particular, higher resistance rates observed in wound-derived isolates suggest that empirical regimens for SSIs may require broader initial coverage than those used for biliary or intra-abdominal fluid isolates, pending culture results.

The mortality rate in our study (23.7%) is higher than in some comparable SSI cohorts, possibly reflecting the severity of underlying surgical pathology (e.g., Fournier’s gangrene, perforated gastric ulcers) and the presence of MDR phenotypes [27,28]. This is in keeping with previous findings that MDR *K. pneumoniae* infections are independently associated with increased mortality, even after adjusting for comorbidities and severity of illness [14,29,30]. Moreover, biofilm formation, a well-described virulence factor in *K. pneumoniae* [31,32], may have contributed to treatment failures in our cohort, as biofilms protect bacteria from both host immunity and antimicrobial penetration. Due to the limited sample size, formal stratified analyses according to ICU admission, MDR status, or specific resistance phenotypes were not feasible and represent an important limitation of this study.

While hypervirulent *K. pneumoniae* (hvKp) strains were not specifically identified in our study, their emergence in surgical populations is an increasing concern [33,34]. HvKp can cause aggressive, metastatic infections even in healthy individuals, and some recent reports describe convergence of hypervirulence and carbapenem resistance, posing a dual threat to patient outcomes [33,35]. Future work should integrate molecular typing to detect such high-risk clones. No molecular virulence testing or phenotypic assays (e.g., string test) were performed; therefore, hvKp could not be systematically identified in this cohort.

From a practical standpoint, these findings support the need for unit-specific antibiograms to guide empirical therapy in surgical infections. Routine reliance on third-generation cephalosporins or fluoroquinolones may be inadequate in similar settings, and early escalation strategies should be informed by local resistance data rather than generalized guidelines alone.

Our study has several limitations. The single-center, retrospective design limits generalisability, and the lack of molecular testing precludes confirmation of specific resistance genes. Furthermore, our dataset may underrepresent polymicrobial infections, as cultures were processed with a focus on clinically dominant pathogens. Nevertheless, the present findings have important implications for surgical infection management. Empirical therapy in our setting should consider the high prevalence of ESBL phenotypes and resistance to commonly used β-lactams and fluoroquinolones, reserving carbapenems and amikacin for confirmed or strongly suspected MDR infections. Infection prevention strategies, including strict perioperative asepsis, antimicrobial stewardship, and targeted surveillance cultures in high-risk patients, are warranted to preserve the efficacy of last-line agents. The absence of molecular or phenotypic virulence testing precluded formal identification of hypervirulent *Klebsiella pneumoniae* strains. AST availability was not uniform across antibiotics and specimen types, which may influence subgroup estimates in smaller categories. The availability of complete clinical data for only a subset of patients limited patient-level analyses but did not affect isolate-based resistance assessments. Incomplete coverage of confirmatory ESBL and carbapenemase testing, as well as the absence of time-trend analysis, represent additional limitations, as pooled resistance rates may mask recent changes in antimicrobial susceptibility.

What is more, colistin susceptibility testing is method-dependent, and automated systems may be unreliable. Broth microdilution is considered the reference method. If broth microdilution was not routinely used in our laboratory, colistin resistance may be over- or under-estimated and should be interpreted cautiously. Given the known limitations of automated methods for colistin susceptibility testing, the reported non-susceptibility rate should be interpreted with caution and confirmed by reference methods in future studies.

Isolate-level line-list susceptibility data are not publicly provided. However, all analyses were conducted at the isolate level, and aggregate resistance phenotypes are transparently reported.

From a practical perspective, all of these findings suggest that empirical use of third-generation cephalosporins or ampicillin–sulbactam may be suboptimal in our surgical setting. In patients with severe infection, prior antibiotic exposure, or biliary and wound-related infections, broader initial coverage may be warranted until susceptibility results are available. Local resistance surveillance should therefore directly inform empirical antibiotic protocols in surgical wards.

## 5. Conclusions

This retrospective study provides a detailed snapshot of *Klebsiella* spp. infections in a general surgery department, highlighting the predominance of surgical site infections, intra-abdominal collections, and biliary tract infections. Our findings reveal a concerning resistance profile, with high rates of non-susceptibility to third-generation cephalosporins, fluoroquinolones, and β-lactam/β-lactamase inhibitor combinations, and frequent ESBL phenotypes, with reported colistin non-susceptibility, which should be interpreted cautiously because of methodological limitations in retrospective susceptibility reporting. This finding should be confirmed with reference colistin testing methods in future prospective surveillance. Encouragingly, susceptibility to carbapenems and amikacin remains largely preserved in our setting, offering effective therapeutic options for severe cases.

The observed 23.7% mortality rate, higher than that reported in some comparable SSI cohorts, likely reflects the severity of underlying surgical pathology and the impact of MDR strains. Resistance patterns varied by infection site, with particularly high scores in cholecystitis and perianal abscesses, underscoring the importance of site-specific resistance surveillance. The potential role of virulence factors, such as biofilm formation, and the looming threat of hypervirulent *K. pneumoniae* strains, further complicate the clinical picture and warrant ongoing vigilance.

Our results reinforce the need for robust local antimicrobial stewardship and infection prevention measures, tailored to the specific epidemiology of surgical wards. They should be interpreted in light of the study’s retrospective, single-center design and the absence of molecular resistance characterization. Empirical regimens should be guided by regular susceptibility data, reserving carbapenems and aminoglycosides for high-risk or confirmed MDR infections. Given the global rise in carbapenem resistance, proactive containment strategies are critical to preserving the efficacy of these last-line agents.

Empirical antibiotic strategies in surgical patients should be guided by updated local susceptibility data rather than generalized recommendations. Future research should include molecular characterization of resistance determinants and virulence genes, enabling earlier recognition of high-risk clones such as carbapenem-resistant or hypervirulent *K. pneumoniae*. Such efforts, combined with multidisciplinary infection control programs, will be essential to reducing the burden of these challenging pathogens in surgical settings.

## Figures and Tables

**Table 1:** Antimicrobial resistance rates (%) by specimen type among *Klebsiella* isolates. Percentages represent resistant isolates among tested isolates (R/tested). Clinical characteristics are analyzed at the patient level and reported separately.

Antibiotic	Abscess	Biliary	Wound	Peritoneal
Ampicillin–sulbactam	0.0% (0/1)	–	33.3% (1/3)	–
Ceftriaxone	50.0% (1/2)	–	50.0% (2/4)	–
Ciprofloxacin	27.8% (5/18)	16.7% (1/6)	71.4% (20/28)	30.0% (3/10)
Colistin	13.3% (2/15)	20.0% (1/5)	60.0% (12/20)	25.0% (2/8)
Piperacillin–tazobactam	33.3% (6/18)	33.3% (2/6)	78.6% (22/28)	40.0% (4/10)
Ceftazidime	29.4% (5/17)	0.0% (0/6)	75.0% (21/28)	40.0% (4/10)
Cefepime	33.3% (6/18)	0.0% (0/6)	65.5% (19/29)	30.0% (3/10)
Imipenem	29.4% (5/17)	0.0% (0/6)	66.7% (18/27)	20.0% (2/10)
Meropenem	35.3% (6/17)	16.7% (1/6)	67.9% (19/28)	30.0% (3/10)
Ertapenem	100.0% (1/1)	–	100.0% (1/1)	0.0% (0/1)
Amikacin	20.0% (3/15)	0.0% (0/6)	66.7% (18/27)	20.0% (2/10)
TMP–SMX	29.4% (5/17)	0.0% (0/6)	71.4% (20/28)	40.0% (4/10)

## Data Availability

Data is presented in the article. Any further querries should be addressed to the corresponding author.

## Supplementary Materials

The following supporting information can be downloaded at: https://www.mdpi.com/article/10.3390/microorganisms14040773/s1, Table S1: Distribution of Klebsiella isolates included in the study. Antimicrobial resistance rates by specimen type among Klebsiella isolates. Percentages represent resistant isolates among tested isolates (R/tested). Demographic and clinical characteristics are reported separately at the patient level; Table S2: Resistance phenotype summary; Table S3: Detailed isolate-level antimicrobial susceptibility data.
