# Supplementary material for: Genus Klebsiella: Infections Encountered in a General Surgery Department and Antimicrobial Drugs Susceptibility Patterns
Source: Microorganisms. 2026 Mar 28;14(4):773. doi: 10.3390/microorganisms14040773 (PMC13119183; doi:10.3390/microorganisms14040773)
Supplement: Supplementary file 1 [file microorganisms-14-00773-s001.zip › microorganisms-4083841-supplementary.pdf]

Supplementary Table S1. Distribution of Klebsiella isolates included in the study. Antimicrobial resistance rates by specimen type among Klebsiella isolates. Percentages represent resistant isolates among tested isolates (R/tested). Demographic and clinical characteristics are reported separately at the patient level.

**Characteristic**		**Number of isolates (%)**
**Species**		
Klebsiella pneumoniae		54 (39,1%)
Klebsiella oxytoca		18 (13%)
Klebsiella spp.		66 (47,8%)
**Specimen type**	**Percentage (%)**	**Approx. number of isolates (n)***
Surgical wound secretions	26.3%	36
Abscess fluid	23.7%	33
Peritoneal fluid	15.8%	22
Biliary fluid	15.8%	22
Other / not specified	18.4%	25
**Total**	**100%**	**138**

Supplementary Table S2. Resistance phenotype summary

**Antimicrobial agent**	**Resistance proportion**	**Resistant isolates (approx. n)***
Ampicillin	High (qualitative)	Not numerically specified
Piperacillin–tazobactam	High (qualitative)	Not numerically specified
Ceftriaxone	High (qualitative)	Not numerically specified
Ciprofloxacin	High non-susceptibility	Not numerically specified
**Colistin**	High non-susceptibility	Not numerically specified
Trimethoprim–sulfamethoxazole	0.12 (12%)	~17
Cefepime	0.12 (12%)	~17

Antimicrobial agent	Resistance proportion	Resistant isolates (approx. n)*
**Imipenem**	0.15 (15%)	~21
**Meropenem**	0.061 (6.1%)	~8
**Ertapenem**	0.00 (0%)	0
**Amikacin**	0.067 (6.7%)	~9

Resistance proportions are derived from pooled isolate-level antimicrobial susceptibility testing results, as presented in the Results and Figure 5. Colistin results represent reported laboratory non-susceptibility and should be interpreted cautiously. Detailed isolate-level antimicrobial susceptibility data are provided in Supplementary Table S3.

Isolate_ID	Patient_ID	Specimen	Infection_Type	Species	AMP	SAM	TZP	CRO	CIP	COL	IPM	MEM	ETP	AMK	SXT	ESBL
1	NA	NA	NA	Klebsiella spp	NA	NA	R	NA	R	NA	R	R	R	R	R	NA
2	NA	NA	NA	Klebsiella spp	NA	NA	R	NA	R	NA	R	R	NA	R	R	NA
3	NA	NA	NA	Klebsiella pneumoniae	NA	NA	R	NA	R	R	R	R	NA	R	R	NA
4	NA	NA	NA	Klebsiella sp	NA	R	R	R	NA	NA	NA	R	NA	S	R	NA
5	P01	wound secretion	foot gangrene	Klebsiella sp	NA	S	S	S	S	NA	S	NA	NA	NA	S	NA
6	P02	peritoneal fluid	peritonitis	Klebsiella pneumoniae	NA	NA	I	NA	S	NA	S	S	NA	S	S	NA
7	NA	NA	NA	Klebsiella pneumoniae	NA	NA	R	NA	R	I	I	R	NA	I	R	NA
8	P03	abscess	liver abscess	Klebsiella pneumoniae ssp pneumoniae	NA	NA	S	NA	S	I	S	S	NA	S	S	NA
9	NA	NA	NA	Klebsiella pneumoniae ssp pneumoniae	NA	NA	S	NA	S	I	S	S	NA	S	S	NA
10	P04	biliary fluid	biliary fluid	Klebsiella spp	NA	NA	S	NA	S	NA	S	S	NA	S	S	NA
11	P05	abscess	facial abscess	Klebsiella sp	NA	S	S	S	S	NA	S	NA	NA	NA	S	NA
12	P06	peritoneal fluid	peritonitis	Klebsiella pneumoniae	NA	NA	S	NA	S	I	S	S	NA	S	R	NA
13	NA	NA	NA	Klebsiella pneumoniae ssp pneumoniae	NA	NA	S	NA	S	I	S	S	NA	S	S	NA
14	P07	liver abscess	liver abscess	Klebsiella pneumoniae ssp pneumoniae	NA	NA	S	NA	S	I	S	S	NA	S	S	NA
15	P08	abscess	flegmon	Klebsiella pneumoniae ssp pneumoniae	R	NA	R	R	R	NA	R	R	R	NA	R	R
16	NA	NA	NA	Klebsiella pneumoniae ssp pneumoniae	NA	NA	R	NA	R	S	R	R	NA	R	R	NA
17	P09	peritoneal fluid	pelvic floor abscess	Klebsiella spp	R	NA	S	NA	S	NA	S	S	S	S	S	NA
18	NA	NA	NA	Klebsiella sp	NA	S	S	S	S	NA	NA	S	NA	S	S	NA
19	NA	NA	NA	Klebsiella pneumoniae	NA	NA	NA	NA	NA	NA	NA	NA	NA	NA	NA	NA
20	NA	NA	NA	Klebsiella pneumoniae	NA	NA	R	NA	R	R	R	R	NA	R	R	NA
21	NA	NA	NA	Klebsiella pneumoniae	NA	NA	R	NA	R	NA	R	R	NA	R	R	NA
22	NA	NA	NA	Klebsiella pneumoniae	NA	NA	NA	NA	NA	NA	NA	NA	NA	NA	NA	NA
23	NA	NA	NA	Klebsiella pneumoniae	NA	NA	R	NA	R	R	S	R	NA	S	S	NA
24	NA	NA	NA	Klebsiella pneumoniae ssp pneumoniae	NA	NA	R	NA	R	S	S	S	NA	R	R	NA
25	NA	NA	NA	Klebsiella pneumoniae ssp pneumoniae	NA	NA	R	NA	R	R	R	R	NA	R	R	NA

26NA	NA	NA	Klebsiella pneumoniae ssp pneumoniae	NA	NA	R	NA	R	R	R	R	NA	R	R	NA
27P10	abscess	ischio-rectal flegmon	Klebsiella pneumoniae	NA	NA	S	NA	S	S	S	S	NA	S	S	NA
28P11	abscess	right buttock abscess	Klebsiella pneumoniae ssp pneumoniae	NA	NA	S	NA	S	I	S	S	NA	S	S	NA
29NA	NA	NA	Klebsiella pneumoniae ssp pneumoniae	NA	NA	R	NA	I	I	R	R	NA	I	R	NA
30NA	NA	NA	Klebsiella pneumoniae ssp pneumoniae	NA	NA	S	NA	S	I	S	S	NA	S	S	NA
31P12	abscess	ischio-rectal flegmon	Klebsiella pneumoniae	NA	NA	S	NA	S	S	S	S	NA	S	S	NA
32NA	NA	NA	Klebsiella pneumoniae ssp pneumoniae	NA	NA	R	NA	R	R	R	R	NA	R	R	NA
33NA	NA	NA	Klebsiella pneumoniae	NA	NA	S	NA	S	S	S	S	NA	S	S	NA
34NA	NA	NA	Klebsiella oxytoca	NA	NA	S	NA	S	S	S	S	NA	S	S	NA
35NA	NA	NA	Klebsiella oxytoca	NA	NA	S	NA	S	S	S	S	NA	S	S	NA
36P13	wound secretion	foot gangrene	Klebsiella pneumoniae	NA	NA	R	NA	R	NA	R	R	NA	NA	R	NA
37NA	NA	NA	Klebsiella pneumoniae	NA	NA	R	NA	R	S	R	R	NA	R	R	NA
38P14	wound secretion	infected thigh hematoma	Klebsiella pneumoniae	NA	NA	NA	NA	S	R	S	S	NA	R	S	NA
39NA	NA	NA	Klebsiella pneumoniae	NA	NA	R	NA	R	R	R	R	NA	R	R	NA
40NA	NA	NA	Klebsiella pneumoniae	NA	NA	R	NA	R	S	NA	R	NA	S	NA	NA
41NA	NA	NA	Klebsiella pneumoniae	NA	NA	NA	NA	R	NA	R	R	NA	R	NA	NA
42NA	NA	NA	Klebsiella pneumoniae	NA	NA	R	NA	R	R	R	R	NA	R	R	NA
43NA	NA	NA	Klebsiella pneumoniae	NA	NA	R	NA	R	R	R	R	NA	R	R	NA
44NA	NA	NA	Klebsiella pneumoniae	NA	NA	R	NA	R	S	R	I	NA	S	R	NA
45NA	NA	NA	Klebsiella pneumoniae	NA	NA	S	NA	S	S	S	S	NA	S	S	NA
46NA	NA	NA	Klebsiella pneumoniae	NA	NA	S	NA	S	S	S	S	NA	S	S	NA
47NA	NA	NA	Klebsiella pneumoniae	NA	NA	R	NA	R	R	NA	I	NA	R	NA	NA
48P15	wound secretion	right liver abscess	Klebsiella pneumoniae	NA	NA	S	NA	S	S	S	S	NA	S	S	NA
49NA	NA	NA	Klebsiella pneumoniae ssp pneumoniae	NA	NA	NA	NA	NA	NA	NA	NA	NA	NA	NA	NA
50P16	wound secretion	foot gangrene	Klebsiella oxytoca	NA	NA	S	NA	I	S	S	S	NA	S	S	NA
51P17	wound secretion	left thigh diabetic gangrene	Klebsiella pneumoniae	NA	NA	R	R	R	NA	R	R	NA	R	NA	NA

52P18	abscess	liver abscess	Klebsiella pneumoniae ssp pneumoniae	NA	NA	S	NA	S	S	S	S	NA	S	S	NA
53P19	abscess	infected liver hematoma	Klebsiella pneumoniae ssp pneumoniae	NA	NA	S	NA	S	S	S	S	NA	S	S	NA
54NA	NA	NA	Klebsiella pneumoniae	NA	NA	S	NA	S	S	S	S	NA	S	S	NA
55P20	biliary fluid	acute cholecistitis	Klebsiella pneumoniae ssp pneumoniae	NA	NA	S	NA	S	S	S	S	NA	S	S	NA
56P21	biliary fluid	acute cholecistitis	Klebsiella pneumoniae	NA	NA	S	NA	S	S	S	S	NA	S	S	NA
57P22	peritoneal fluid	peritonitis	Klebsiella pneumoniae ssp pneumoniae	NA	NA	R	NA	R	R	R	R	NA	R	R	NA
58NA	NA	NA	Klebsiella pneumoniae	NA	NA	R	NA	R	R	R	R	NA	R	R	NA
59NA	NA	NA	Klebsiella pneumoniae ssp pneumoniae	NA	NA	R	NA	R	R	R	R	NA	R	R	NA
60NA	NA	NA	Klebsiella pneumoniae ssp pneumoniae	NA	NA	R	NA	R	R	R	R	NA	R	R	NA
61NA	NA	NA	Klebsiella pneumoniae ssp pneumoniae	NA	NA	R	NA	R	R	R	R	NA	R	R	NA
62NA	NA	NA	Klebsiella pneumoniae ssp pneumoniae	NA	NA	S	NA	S	S	S	S	NA	S	S	NA
63NA	NA	NA	Klebsiella pneumoniae	NA	NA	R	NA	S	S	S	S	NA	S	S	NA
64NA	NA	NA	Klebsiella pneumoniae ssp pneumoniae	NA	NA	R	NA	R	R	R	R	NA	S	R	NA
65P23	pus	FLEGMON FOSA ISCHIORECTALA	Klebsiella pneumoniae	NA	NA	S	NA	S	S	S	S	NA	S	S	NA
66P24	abscess	ABCESE HEPATICE MULTIPLE RECIDIVATE LHD	Klebsiella pneumoniae	NA	NA	S	NA	S	S	S	S	NA	S	S	NA
67NA	NA	NA	Klebsiella pneumoniae	NA	NA	R	NA	R	R	R	R	NA	R	R	NA
68P25	biliary fluid	cholecistitis	Klebsiella pneumoniae	NA	NA	R	NA	S	S	S	S	NA	S	S	NA
69NA	NA	NA	Klebsiella pneumoniae	NA	NA	S	NA	R	S	S	S	NA	S	R	NA
70NA	NA	NA	Klebsiella pneumoniae	NA	NA	R	NA	S	S	S	S	NA	S	S	NA
71NA	NA	NA	Klebsiella pneumoniae	NA	NA	R	NA	S	NA	S	S	NA	S	S	NA
72NA	NA	NA	Klebsiella pneumoniae	NA	NA	R	NA	R	S	R	R	NA	R	R	NA
73NA	NA	NA	Klebsiella pneumoniae	NA	NA	R	NA	R	R	R	R	NA	R	R	NA
74NA	NA	NA	Klebsiella pneumoniae	NA	NA	R	NA	R	R	R	R	NA	R	R	NA

75	NA	NA	NA	Klebsiella oxytoca	NA	NA	R	NA	R	NA	NA	R	NA	NA	NA	NA
76	NA	NA	NA	Klebsiella spp	NA	NA	R	NA	R	NA	R	R	R	R	R	NA
77	NA	NA	NA	Klebsiella spp	NA	NA	R	NA	R	NA	R	R	NA	R	R	NA
78	NA	NA	NA	Klebsiella pneumoniae	NA	NA	R	NA	R	R	R	R	NA	R	R	NA
79	NA	NA	NA	Klebsiella sp	NA	R	R	R	NA	NA	NA	R	NA	S	R	NA
80	P01	wound secretion	left hallux gangrene	Klebsiella sp	NA	S	S	S	S	NA	S	NA	NA	NA	S	NA
81	P02	peritoneal fluid	acute peritonitis	Klebsiella pneumoniae	NA	NA	I	NA	S	NA	S	S	NA	S	S	NA
82	NA	NA	NA	Klebsiella pneumoniae	NA	NA	R	NA	R	I	I	R	NA	I	R	NA
83	P03	abscess	liver abscess	Klebsiella pneumoniae ssp pneumoniae	NA	NA	S	NA	S	I	S	S	NA	S	S	NA
84	NA	NA	NA	Klebsiella pneumoniae ssp pneumoniae	NA	NA	S	NA	S	I	S	S	NA	S	S	NA
85	P04	biliary fluid	cholecistitis	Klebsiella spp	NA	NA	S	NA	S	NA	S	S	NA	S	S	NA
86	P05	abscess	left foot abscess	Klebsiella sp	NA	S	S	S	S	NA	S	NA	NA	NA	S	NA
87	P06	peritoneal fluid	peritonitis	Klebsiella pneumoniae	NA	NA	S	NA	S	I	S	S	NA	S	R	NA
88	NA	NA	NA	Klebsiella pneumoniae ssp pneumoniae	NA	NA	S	NA	S	I	S	S	NA	S	S	NA
89	P07	liver abscess	liver abscess	Klebsiella pneumoniae ssp pneumoniae	NA	NA	S	NA	S	I	S	S	NA	S	S	NA
90	P08	abscess	flegmon	Klebsiella pneumoniae ssp pneumoniae	R	NA	R	R	R	NA	R	R	R	NA	R	R
91	NA	NA	NA	Klebsiella pneumoniae ssp pneumoniae	NA	NA	R	NA	R	S	R	R	NA	R	R	NA
92	P09	peritoneal fluid	pelvic floor abscess	Klebsiella spp	R	NA	S	NA	S	NA	S	S	S	S	S	NA
93	NA	NA	NA	Klebsiella sp	NA	S	S	S	S	NA	NA	S	NA	S	S	NA
94	NA	NA	NA	Klebsiella pneumoniae	NA	NA	NA	NA	NA	NA	NA	NA	NA	NA	NA	NA
95	NA	NA	NA	Klebsiella pneumoniae	NA	NA	R	NA	R	R	R	R	NA	R	R	NA
96	NA	NA	NA	Klebsiella pneumoniae	NA	NA	R	NA	R	NA	R	R	NA	R	R	NA
97	NA	NA	NA	Klebsiella pneumoniae	NA	NA	NA	NA	NA	NA	NA	NA	NA	NA	NA	NA
98	NA	NA	NA	Klebsiella pneumoniae	NA	NA	R	NA	R	R	S	R	NA	S	S	NA
99	NA	NA	NA	Klebsiella pneumoniae ssp pneumoniae	NA	NA	R	NA	R	S	S	S	NA	R	R	NA
100	NA	NA	NA	Klebsiella pneumoniae ssp pneumoniae	NA	NA	R	NA	R	R	R	R	NA	R	R	NA

101NA	NA	NA	Klebsiella pneumoniae ssp pneumoniae	NA	NA	R	NA	R	R	R	R	NA	R	R	NA
102P10	abscess	pelvic floor abscess	Klebsiella pneumoniae	NA	NA	S	NA	S	S	S	S	NA	S	S	NA
103P11	abscess	left buttock abscess	Klebsiella pneumoniae ssp pneumoniae	NA	NA	S	NA	S	I	S	S	NA	S	S	NA
104NA	NA	NA	Klebsiella pneumoniae ssp pneumoniae	NA	NA	R	NA	I	I	R	R	NA	I	R	NA
105NA	NA	NA	Klebsiella pneumoniae ssp pneumoniae	NA	NA	S	NA	S	I	S	S	NA	S	S	NA
106P12	abscess	ischio-rectal flegmon	Klebsiella pneumoniae	NA	NA	S	NA	S	S	S	S	NA	S	S	NA
107NA	NA	NA	Klebsiella pneumoniae ssp pneumoniae	NA	NA	R	NA	R	R	R	R	NA	R	R	NA
108NA	NA	NA	Klebsiella pneumoniae	NA	NA	S	NA	S	S	S	S	NA	S	S	NA
109NA	NA	NA	Klebsiella oxytoca	NA	NA	S	NA	S	S	S	S	NA	S	S	NA
110NA	NA	NA	Klebsiella oxytoca	NA	NA	S	NA	S	S	S	S	NA	S	S	NA
111P13	wound secretion	right hallux gangrene	Klebsiella pneumoniae	NA	NA	R	NA	R	NA	R	R	NA	NA	R	NA
112NA	NA	NA	Klebsiella pneumoniae	NA	NA	R	NA	R	S	R	R	NA	R	R	NA
113P14	wound secretion	infected thigh hematoma	Klebsiella pneumoniae	NA	NA	NA	NA	S	R	S	S	NA	R	S	NA
114NA	NA	NA	Klebsiella pneumoniae	NA	NA	R	NA	R	R	R	R	NA	R	R	NA
115NA	NA	NA	Klebsiella pneumoniae	NA	NA	R	NA	R	S	NA	R	NA	S	NA	NA
116NA	NA	NA	Klebsiella pneumoniae	NA	NA	NA	NA	R	NA	R	R	NA	R	NA	NA
117NA	NA	NA	Klebsiella pneumoniae	NA	NA	R	NA	R	R	R	R	NA	R	R	NA
118NA	NA	NA	Klebsiella pneumoniae	NA	NA	R	NA	R	R	R	R	NA	R	R	NA
119NA	NA	NA	Klebsiella pneumoniae	NA	NA	R	NA	R	S	R	I	NA	S	R	NA
120NA	NA	NA	Klebsiella pneumoniae	NA	NA	S	NA	S	S	S	S	NA	S	S	NA
121NA	NA	NA	Klebsiella pneumoniae	NA	NA	S	NA	S	S	S	S	NA	S	S	NA
122NA	NA	NA	Klebsiella pneumoniae	NA	NA	R	NA	R	R	NA	I	NA	R	NA	NA
123P15	wound secretion	foot gangrene	Klebsiella pneumoniae	NA	NA	S	NA	S	S	S	S	NA	S	S	NA
124NA	NA	NA	Klebsiella pneumoniae ssp pneumoniae	NA	NA	NA	NA	NA	NA	NA	NA	NA	NA	NA	NA
125P16	wound secretion	foot gangrene	Klebsiella oxytoca	NA	NA	S	NA	I	S	S	S	NA	S	S	NA
126P17	wound secretion	diabetic gangrene	Klebsiella pneumoniae	NA	NA	R	R	R	NA	R	R	NA	R	NA	NA
127P18	abscess	liver abscess	Klebsiella pneumoniae ssp pneumoniae	NA	NA	S	NA	S	S	S	S	NA	S	S	NA

128P19	abscess	infected liver hematoma	Klebsiella pneumoniae ssp pneumoniae	NA	NA	S	NA	S	S	S	S	NA	S	S	NA
129NA	NA	NA	Klebsiella pneumoniae	NA	NA	S	NA	S	S	S	S	NA	S	S	NA
130P20	biliary fluid	cholecistitis	Klebsiella pneumoniae ssp pneumoniae	NA	NA	S	NA	S	S	S	S	NA	S	S	NA
131P21	biliary fluid	cholecistitis	Klebsiella pneumoniae	NA	NA	S	NA	S	S	S	S	NA	S	S	NA
132P22	peritoneal fluid	peritonitis	Klebsiella pneumoniae ssp pneumoniae	NA	NA	R	NA	R	R	R	R	NA	R	R	NA
133NA	NA	NA	Klebsiella pneumoniae	NA	NA	R	NA	R	R	R	R	NA	R	R	NA
134NA	NA	NA	Klebsiella pneumoniae ssp pneumoniae	NA	NA	R	NA	R	R	R	R	NA	R	R	NA
135NA	NA	NA	Klebsiella pneumoniae ssp pneumoniae	NA	NA	R	NA	R	R	R	R	NA	R	R	NA
136NA	NA	NA	Klebsiella pneumoniae ssp pneumoniae	NA	NA	R	NA	R	R	R	R	NA	R	R	NA
137NA	NA	NA	Klebsiella pneumoniae ssp pneumoniae	NA	NA	S	NA	S	S	S	S	NA	S	S	NA
138NA	NA	NA	Klebsiella pneumoniae	NA	NA	R	NA	S	S	S	S	NA	S	S	NA

Abbreviations: AMP = ampicillin; SAM = ampicillin–sulbactam; TZP = piperacillin–tazobactam; CRO = ceftriaxone; CIP = ciprofloxacin; COL = colistin; IPM = imipenem; MEM = meropenem; ETP = ertapenem; AMK = amikacin; SXT = trimethoprim–sulfamethoxazole. S = susceptible; I = intermediate; R = resistant; NA = not available.
